# Differences in Inflammatory Marker Kinetics between the First and Second Wave of COVID-19 Patients Admitted to the ICU: A Retrospective, Single-Center Study

**DOI:** 10.3390/jcm10153290

**Published:** 2021-07-26

**Authors:** Tamas Szakmany, William Tuckwell, Elsa Harte, Nick Wetherall, Saraswathi Ramachandran, Shannon Price, Henry Breen, Charlotte Killick, Yusuf Cheema, Charles King, Owen Richards

**Affiliations:** 1 Critical Care Directorate, Grange University Hospital, Aneurin Bevan University Health Board, Llanyravon, Cwmbran NP44 8YN, UK; 2 Department of Anaesthesia, Intensive Care and Pain Medicine, Division of Population Medicine, Cardiff University, Cardiff CF14 4XN, UK; TuckwellWG@cardiff.ac.uk (W.T.); HarteE1@cardiff.ac.uk (E.H.); wetherallNP@cardiff.ac.uk (N.W.); RamachandranS1@cardiff.ac.uk (S.R.); PriceSM2@cardiff.ac.uk (S.P.); BreenH1@cardiff.ac.uk (H.B.); killickcj@cardiff.ac.uk (C.K.); CheemaYA@cardiff.ac.uk (Y.C.); kingc20@cardiff.ac.uk (C.K.); RichardsO3@cardiff.ac.uk (O.R.)

**Keywords:** COVID-19, procalcitonin, C-reactive protein, corticosteroid, immunomodulation

## Abstract

Background: We sought to determine if there was a difference in the longitudinal inflammatory response measured by white blood cell count (WBC), C-reactive protein (CRP), procalcitonin (PCT), and ferritin levels between the first and the second COVID-19 wave of ICU patients. Methods: In a single-center retrospective observational study, ICU patients were enrolled during the first and second waves of the COVID-19 pandemic. Data were collected on patient demographics, comorbidities, laboratory results, management strategies, and complications during the ICU stay. The inflammatory response was evaluated using WBC count, CRP, PCT, and Ferritin levels on the day of admission until Day 28, respectively. Organ dysfunction was measured by the SOFA score. Results: 65 patients were admitted during the first and 113 patients during the second wave. WBC and ferritin levels were higher in the second wave. CRP and PCT showed markedly different longitudinal kinetics up until day 28 of ICU stay between the first and second wave, with significantly lower levels in the second wave. Steroid and immunomodulatory therapy use was significantly greater in the second wave. Mortality was similar in both waves. Conclusions: We found that there was a significantly reduced inflammatory response in the second wave, which is likely to be attributable to the more widespread use of immunomodulatory therapies.

## 1. Introduction

A novel coronavirus disease (COVID-19) is caused by severe acute respiratory syndrome coronavirus 2 (SARS-CoV-2) and is responsible for the current global pandemic declared by the World Health Organisation (WHO) on 11th March 2020 [1]. The disease most commonly manifests as a respiratory illness, causing pneumonia and acute respiratory distress syndrome (ARDS) in those patients most affected by it, and has resulted in millions of deaths worldwide [2]. 

Host-mediated lung and other tissue inflammation is present and drives the mortality in severe COVID-19 [3]. Studies following the clinical course of COVID-19 established a strong connection between the severity of clinical presentation and inflammatory cell infiltration, as well as the amplification of cytokine release [4]. Elevated levels of C-reactive protein (CRP) and Procalcitonin (PCT) have been associated with severe COVID-19, which suggests that these can be used as biomarkers for disease prognosis [5]. Increased white blood cell count (WBC) has been established as being indicative of inflammation and has also been associated with increased mortality in COVID-19 [6]. PCT measurement has been rapidly adopted by UK hospitals and intensive care units during the first wave of the pandemic; however, longitudinal data are still sparse [7,8]. Likewise, early on in the pandemic, elevated ferritin levels were reported and implicated in the development of the so-called cytokine storm [9]. Ferritin synthesis can be stimulated by several inflammatory cytokines, including interleukin-6 (IL-6), which has been specifically targeted in COVID-19 [10].

Since the beginning of the pandemic, low-dose corticosteroids and more recently IL-6 receptor inhibitors have been shown to improve outcomes in hospitalized patients with organ dysfunction [11,12,13]. The rapid adoption of these treatments led to a different patient case-mix on the ICUs between the first and second waves [14]. It is unclear how this change has been manifested in the longitudinal inflammatory response. 

### Aims and Objectives

The objective of this study was to determine if there was a difference in the longitudinal inflammatory response measured by WBC, CRP, PCT, and ferritin levels between the first COVID-19 wave and the second COVID-19 wave of ICU patients. 

## 2. Materials and Methods

This was a single-center retrospective observational study carried out in Aneurin Bevan University Health Board in Wales, United Kingdom. Patients were enrolled during the first and second wave of the COVID19 pandemic (first wave: 9 March 2020 to 5 June 2020, second wave: 17 November 2020 to 15 March 2021). 

During the study, consecutive patients admitted to the ICU were screened daily and recruited to the study if they fulfilled all inclusion criteria: Age ≥ 18-years old; SARS-CoV-2 infection confirmed by positive RT-PCR. Data were collected on patient demographics, comorbidities, physiological and laboratory results, management strategies, and complications during the ICU stay up to 28 days from ICU admission. For outcome ascertainment, patients were followed up until discharge from the hospital.

The inflammatory response was evaluated using the following parameters: White Blood Cell count, CRP, PCT, Ferritin levels on the day of admission (Day 0), Day 3, 7, 10, 14, 21, and 28, respectively. Organ dysfunction was measured by the SOFA score on the same days. We have defined hyperinflammation as a categorical variable if any of the following were present: CRP concentration greater than 150 mg/L; a doubling of C-reactive protein concentration within 24 h from a concentration of greater than 50 mg/L; or a ferritin concentration of greater than 1500 μg/L. These cutoffs were agreed from a literature review and were published previously [15].

Concomitant treatment: We have recorded steroid administration and any other experimental immunomodulatory treatment for each patient [16,17,18]. Low-dose dexamethasone, 6 mg daily for ten days, was administered to every patient from 16th June 2020 onwards as per the recommendation of the Chief Medical Officer of Wales. All eligible patients were recruited to the RECOVERY and REMAP-CAP trials and any experimental treatment was only administered as part of their study allocation. Following the announcement of the REMAP-CAP study results on IL-6 receptor inhibitors on the 8th January 2021, every patient has received 8 mg/kg tocilizumab within 24 h of ICU admission. Antimicrobial treatment was left at clinical discretion and, in general, our ICU has adopted the guidance described by the consensus panel on PCT use earlier [19].

Patients were admitted to the ICU following discussion with at least two senior decision makers in both waves. Our hospital run a respiratory high-dependency area developed during the first wave and deployed in the second wave, offering non-invasive respiratory support, including CPAP and high-flow nasal oxygen therapy. Most of our patients therefore were admitted for mechanical ventilation as they failed to progress on conventional oxygen support and non-invasive ventilation.

Data management, monitoring, and reporting of the study were performed in accordance with the Good Clinical Practice Guidelines. The study was registered with the Research and Development Department at Aneurin Bevan University Health Board and internal risk review classed it as a service evaluation, with no formal ethical approval or informed consent required, due to the anonymized retrospective data collection of routine clinical data (PICOT: Evaluation of Procalcitonin Measurement in COVID Patients Admitted to ICU. ABUHB Risk Review Committee, 28 April 2020)

### Statistical Analysis

Categorical variables are described as proportions and are compared using Fisher’s exact test. Continuous variables are described as median (inter-quartile range) and compared using a Mann-Whitney U test between the groups. We used the multivariate mixed general linear model to describe changes in biomarkers between the groups and during the study period. Approximately 10% of the patients had missing values in the inflammatory markers in the cohort, and we have used group medians for imputing values. A post hoc analysis of survivors vs. non-survivors were carried out in both groups to understand any differences between biomarker kinetics. A two-tailed *p*-value < 0.05 was considered statistically significant. All statistical tests were calculated using SPSS 26.0 (SPSS Inc., Chicago, IL, USA).

## 3. Results

During the observation periods 178 patients were admitted to the ICU with a positive SARS-CoV-2 PCR test and recruited into the study. Sixty-five patients were admitted during the first wave and 113 patients were admitted during the second wave. All patients were followed up until hospital discharge. Demographic data, baseline characteristics, and treatment modalities used are presented in Table 1. 

As expected, due to policy change, all patients in the second wave received low-dose corticosteroids and 75 (66.4%) of them received IL-6 and IL-1 receptor inhibitors. Patients in the second wave were significantly older and had more cardiovascular and other comorbidities. They spent significantly shorter periods in the ICU. The number of patients at risk in the two waves on each day of observation is presented in Table S1. There was no statistically significant difference in hospital mortality between the two waves. 

**Figure 1:**
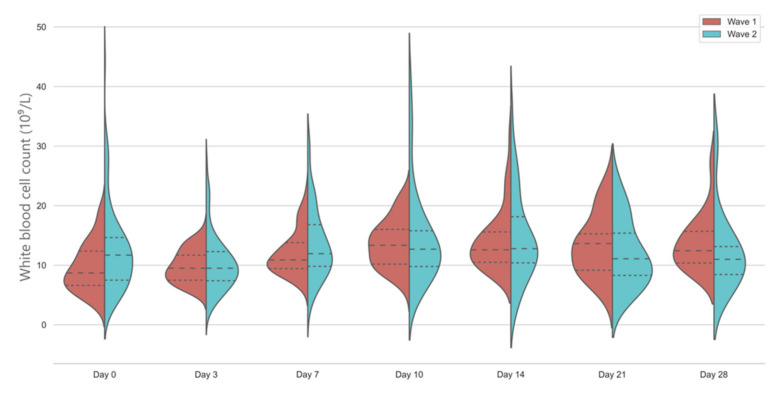
Temporal changes in white blood cell counts in the first and second wave. Violin plots represent the distribution of data. Dashed lines show medians and interquartile ranges. The general linear model indicates significant differences between days in wave 1 and between groups, *p* < 0.001.

White blood cell count showed a significant increase from baseline in the first wave; however, there was no significant longitudinal change in the second wave. Median WCC was slightly above normal in the second wave on the day of admission, whilst it was well within the normal range in the first wave (Figure 1).

**Figure 2:**
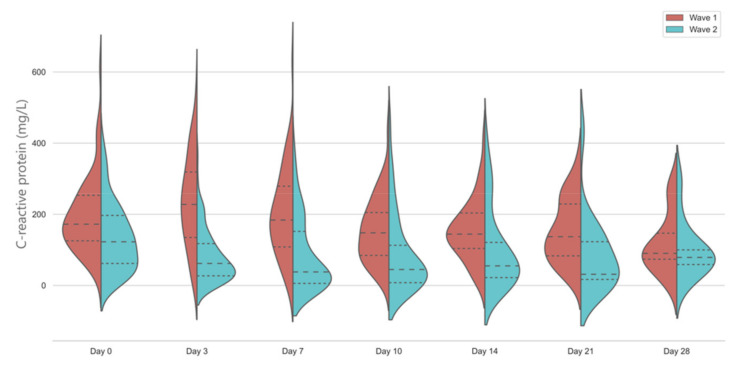
Temporal changes in C-reactive protein levels in the first and second wave. Violin plots represent distribution of data. Dashed lines show medians and interquartile ranges. The general linear model indicates significant differences between days in wave 1 and between groups, *p* < 0.001.

Inflammatory protein markers were significantly lower in the second wave, compared to the first wave. CRP levels showed a markedly different kinetic in the first wave, with a significant rise on Day 3 and levelling off after Day 7. In the second wave, CRP levels were significantly lower on admission and continued to decrease further, with a slight rise at Day 14 (Figure 2).

**Figure 3:**
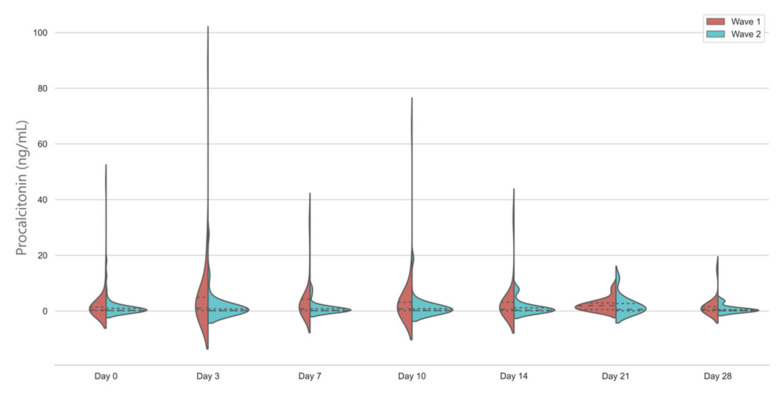
Temporal changes in procalcitonin levels in the first and second wave. Violin plots represent the distribution of data. Dashed lines show medians and interquartile ranges. The general linear model indicates significant differences between days in wave 1 and between groups, *p* < 0.001.

Median PCT levels were below the 0.5 ng/mL threshold on admission on both waves; however, in the first wave, there was a significant and sustained rise throughout the study period. In the second wave, the vast majority of the patients had PCT levels below 0.25 ng/mL throughout the study, with no significant change compared to baseline (Figure 3).

Ferritin levels in the second wave were only measured on Day 0 and Day 3. At both timepoints, levels were significantly lower in the first wave: Day 0: 856 (441–2651) µg/L vs. 1755 (753–2853) µg/L and Day 3: 883 (484–3206) µg/L vs. 1446 (810–3227) µg/L in the first and second wave, *p* = 0.001, respectively. 

Hyperinflammatory phenotype based on CRP and ferritin levels was present in 61 out of 65 (93.8%) patients in the first wave, compared to 74 out of 113 (65.5%) of patients in the second wave (*p* = 0.001). In the first wave, 56 (86.2%) patients were in the hyperinflammatory phenotype group early in the course of their illness (between Day 0 and Day 3) compared to 54 (47.8%) of the patients in the second wave (*p* = 0.001). Mortality was higher in the hyperinflammatory 62/135 (45.9%) than in the hypoinflammatory 13/43 (30.2%) phenotype group, however the difference was not statistically significant (*p* = 0.078). 

**Figure 4:**
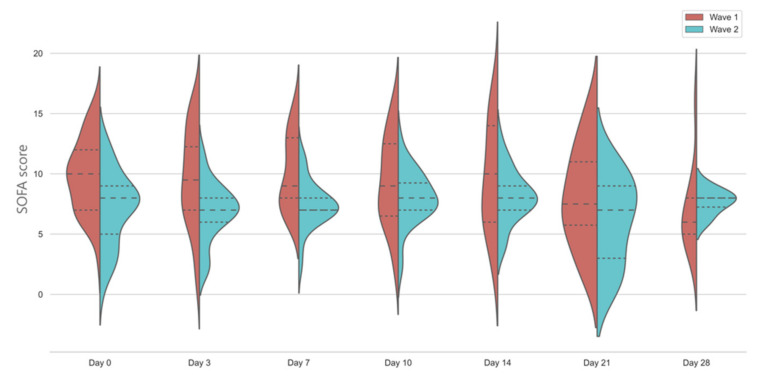
Temporal changes in SOFA scores in the first and second wave. Violin plots represent the distribution of data. Dashed lines show medians and interquartile ranges. The general linear model indicates significant differences between days in wave 1 and between groups, *p* < 0.001.

SOFA scores were significantly higher on Day 0 and Day 3 in the first-wave patients, with no significant change throughout the study period in those patients who survived and still on the ICU by day 28 (Figure 4.). In both waves, SOFA scores indicated sustained multi-organ failure with over three quarters of patients having SOFA > 4.

## 4. Discussion

In our retrospective observational study, we found that commonly measured inflammatory markers such as CRP and PCT had markedly different longitudinal kinetics up until day 28 of ICU stay between the first and second wave of the pandemic. To our knowledge, we are the first to report this significant difference, which can have implications in clinical practice as well as directing future research.

In our retrospective study, we have seen that WBC count was highly variable in patients with severe COVID-19 disease; however, the majority of the patients in the first wave had normal values on admission, with some fluctuation afterwards. Unsurprisingly, a sensitive but highly unspecific laboratory marker of inflammation did not show significant differences. Similar trends in WBC until day 30 of hospitalization were observed previously in patient cohorts from the first wave [15,20]. It is still unclear how low-dose steroid treatment will affect the WBC levels in the context of severe COVID-19 disease; hence, the clinical interpretation of these values is difficult.

CRP and PCT values were significantly different between the two waves and the temporal changes showed a different pattern as well. Our general linear model clearly identified these significant pattern differences, and our data could be used as a benchmark for further, larger studies. The most plausible explanation for the different inflammatory marker trends is the use of evidence-based immunomodulatory therapies in the second wave. Corticosteroid use has been shown to result in a reduction in CRP levels in community-acquired pneumonia [21] and IL-6 and IL-1 receptor antagonists have been also shown to drastically alter the CRP response [22]. Our finding that CRP levels were significantly reduced after the day of ICU admission, when most patients had tocilizumab, sarilumab, or anakinra administered, confirms this previous observation [22]. In our patients, we have observed a modest increase in CRP levels around day 14 of ICU stay. It is possible that this elevation might coincide with nosocomial infections; however, this needs further evaluation of microbiology data. Our results reflect the diagnostic uncertainty the bedside clinicians face when they try to distinguish ongoing inflammation from new bacterial infection. 

An important finding of our study is that PCT, which has been rapidly adopted in the UK ICUs in the hope that its well documented characteristics will help differentiate bacterial infection from viral illness related inflammation, has shown significantly different temporal trends [7]. This was almost completely flat in the second wave, with over 75% of patients having less than 0.5 ng/mL PCT values throughout the study period, implying the low probability of bacterial infection [19]. Further studies are required to elucidate if changes in PCT could be used as an effective biomarker to help antimicrobial stewardship in patients treated with low-dose corticosteroids and Il-6 receptor inhibitors. Our results also show that any PCT data in this regard from the first wave would be difficult to interpret in the current therapeutic context as we have seen significantly higher PCT levels throughout the ICU stay in the patients who were treated with standard care, without immunomodulators.

Contrary to the other two parameters, we found significantly lower ferritin levels in the early period in the first wave. The ferritin levels in that cohort were similar to what has been reported in the UK, including in our neighboring hospitals [23]. The higher observed ferritin levels in the second wave might reflect the higher risk of death the patients experienced, as increasing ferritin levels have been associated with increased mortality in previous studies [24]. It is also possible that the patients admitted in the second wave were more likely in the group, who could be described by the rapidly increasing ferritin levels, which is linked to worse outcomes [24]. Rising ferritin levels have been linked not only to inflammation, but also to direct cellular damage and more organ dysfunction [15]. Our patients in the second wave have spent more time in the hospital and on non-invasive respiratory support before ICU admission (data not shown) therefore it is plausible that they have accrued more cellular and organ damage by the time they were recruited in our study. This could also explain the higher mortality observed in the second wave.

We have previously shown that the so-called hyperinflammatory phenotype of COVID19 ARDS has a similar prevalence to other disease processes [25]. This phenotype has been derived from a parsimonious model utilizing IL-6, soluble tumor necrosis factor receptor and bicarbonate levels and has been validated in previous ARDS trials [26]. In the present study, we have utilized a different classification for characterizing the inflammatory response, based on CRP and ferritin level kinetics based on the work of Manson et al., where the hyperinflammatory phenotype was associated with worse outcomes [15]. Contrasting to their results, significantly higher proportions of our patients were in the hyperinflammatory phenotype group (86.2% vs. 33.3% in the Manson study); however, this is likely to be attributable to the different patient populations, as in the first wave, almost all of our patients were mechanically ventilated, whilst in the previous study, patients were included on hospital admission with no or minimal oxygen support. Therefore, our results support their findings, that the hyperinflammatory group is more likely to need significant respiratory support [15]. Mortality in the hyperinflammatory group was higher, mirroring the results of previous studies, including ours [15,25]. Interestingly, we have found that a significantly lower proportion of patients were in the hyperinflammatory group in the second wave, but with numerically higher mortality. This finding underlines any scoring system developed in the first wave of the pandemic should be recalibrated and revalidated in the new phases, as otherwise their utility and face validity would be questionable in a population with different inflammatory response, but seemingly similar mortality risk [27].

SOFA scores were significantly higher in the first wave on admission, however it is unclear how could we best interpret the reduction in the median SOFA score by 2 points in the second wave. In both cohorts, SOFA scores were in the same range as we have observed previously in patients admitted to the Welsh ICUs following acute hypoxaemic respiratory failure [28]. Contrasting this, we found higher mortality in the second wave. We can only speculate what has caused this phenomenon, which have been observed nationally, especially in the patients needing mechanical ventilation. It is possible that in the second wave, this was a self-selected higher risk population, who failed non-invasive respiratory support, despite the use of disease modifying treatments such as steroids [29]. Our data might also reflect that the patients in the second wave were at a different trajectory of their inflammatory response; however, this needs to be corroborated by a much larger dataset. Importantly, despite much better adherence to ARDS best-practice guidelines in low tidal volume ventilation and more frequent use of prone positioning, the mortality was similar in our COVID-19 cohort to our previous study, but in line with the UK national average [28,29].

There were significant differences in the management of the patients between the first and second wave. In the first wave, we only used steroids as part of the RECOVERY trial, which meant that the time of administration varied. Following the publication of the RECOVERY trial results on dexamethasone and the REMAP-CAP data on IL-6R inhibitors, our unit has rapidly adopted the evidence, which has led to a significant increase in the use of these agents. In the second wave, all our patients had already had steroids administered before ICU admission; however, they were again at various phases of their treatment (data not shown). Most patients received IL-6R blocking agents in the second wave within 24 h of ICU admission but following the Day 0 blood results. Our findings of the significantly different CRP kinetics between the first and second wave from Day 0 to Day 3 probably reflect the effects of these agents.

Our study has significant limitations, mostly due to the retrospective nature of the data collection and due to the missingness of laboratory data in approximately 10% of the patients. We have used established imputing strategies; however, these could still skew the results. Our single-center data might not be generalizable; however, our results are very much in line with reports from the UK and other high-income countries [15,23,24,29]. We have also not been able to report on antimicrobial use and microbiology results, which would give important insight into the usefulness of using CRP and PCT levels as aids for antimicrobial stewardship on the ICU in patients with COVID-19. Due to the small sample size, we have not attempted to develop a predictive model using the variables available, as this is very likely to lead to an overfitted and not generalizable model [27].

## 5. Conclusions

In conclusion, we found that there was a significantly reduced inflammatory response in the second wave, which is likely to be attributable to the more widespread use of immunomodulatory therapies. Patients’ clinical characteristics were unchanged, with higher mortality in the second wave. Our data indicate that we should interpret any data derived from the first part of the pandemic with caution and there are further studies needed to elucidate if the different temporal inflammatory response could be used for prognostic and diagnostic purposes in COVID-19 patients treated with steroids and IL-6 receptor inhibitors.

## Figures and Tables

**Table 1:** Demographics, treatment modalities, and outcomes in COVID-19 patients admitted in the first and second wave.

	First Wave (*n* = 65)	Second Wave (*n* = 113)	*p*-Value
Age (years)	57 (51–63)	61 (53–67)	0.024
Sex (Male/Female, *n*)	43/22	30/83	0.310
Diabetes	18	26	0.589
Hypertension	28	42	0.524
Ischaemic heart disease	3	23	0.004
COPD	1	6	0.425
Asthma	17	23	0.456
Chronic renal disease	2	11	0.137
Other comorbidities	10	42	0.002
Mechanical ventilation	64 (98.5%)	103 (91.2%)	0.883
SOFA score on admission	10 (7–12)	8 (5–9)	0.001
Dexamethasone (*n*, %)	14 (21.5%)	113 (100%)	0.001
Tocilizumab (*n*, %)	2 (3.1%)	63 (55.8%)	0.001
Sarilumab (*n*, %)	0	9 (7.9%)	N/A
Anakinra (*n*, %)	0	3 (2.6%)	N/A
Length of ICU stay (days)	18 (13–30)	8.2 (4–16.7)	0.001
Hospital mortality (*n*, %)	21 (32.3%)	54 (47.8%)	0.058

COPD: Chronic obstructive pulmonary disease; SOFA: Sequential Organ Failure Assessment; ICU: Intensive Care Unit.

## Data Availability

The data presented in this study are available on request from the corresponding author.

## Supplementary Materials

The following are available online at https://www.mdpi.com/article/10.3390/jcm10153290/s1, Table S1: Number of patients at risk on the ICU for each days of observation.
