# Supplementary material for: Differences in Inflammatory Marker Kinetics between the First and Second Wave of COVID-19 Patients Admitted to the ICU: A Retrospective, Single-Center Study
Source: J Clin Med. 2021 Jul 26;10(15):3290. doi: 10.3390/jcm10153290 (PMC8348515; doi:10.3390/jcm10153290)
Supplement: Supplementary file 1 [file jcm-10-03290-s001.zip › jcm-1254454-supplementary.pdf]

Table S1. Number of patients at risk on the ICU for each days of observation.

	Day 0	Day 3	Day 7	Day 10	Day 14	Day 21	Day 28
Wave 1	65	62	58	52	51	25	16
Wave 2	108	87	57	39	30	18	12
